# Room-temperature structure determination of vacuum-sensitive organic compounds by formvar encapsulation and serial electron diffraction

**DOI:** 10.1107/S1600576725009823

**Published:** 2025-11-26

**Authors:** Sreelaja Pulleri Vadhyar, Ehsan Nikbin, Hazem Daoud, Jane Y. Howe, R. J. Dwayne Miller

**Affiliations:** a Department of Chemistry University of Toronto Toronto OntarioM5S3H6 Canada; b Department of Physics University of Toronto Toronto OntarioM5S1A7 Canada; c Department of Materials Science and Engineering University of Toronto Toronto OntarioM5S3E4 Canada; Goa University, India

**Keywords:** structure determination, TEM sample preparation, transmission electron microscopy, electron diffraction, organic molecules, room-temperature diffraction studies, formvar

## Abstract

Samples used in electron microscopy are traditionally required to be stable under vacuum. However, many organic compounds with high vapor pressures readily sublime at room temperature, a process that is further accelerated under the high-vacuum conditions of an electron microscope. Here, we demonstrate for the first time the structure determination of vacuum-sensitive organic compounds in their solid state at room temperature using electron crystallography. Serial electron diffraction was employed to obtain sub-Å-resolution structures of anthracene and pyrene, two representative organic molecules which sublimate under the high-vacuum conditions of a transmission electron microscope. This was made possible by a simple sample preparation technique in which ultrathin crystals are encapsulated with a formvar layer to prevent sublimation under vacuum. By combining serial electron diffraction with formvar encapsulation, we demonstrate high-resolution structure determination of vacuum-sensitive samples at room temperature rather than cryogenic conditions. Moreover, by avoiding low-temperature phase transitions that can alter material properties, this method expands the accessible temperature range for studying the structural characteristics of vacuum-sensitive materials.

## Abstract 2

This article reports sub-Å structure determination of vacuum-sensitive organic samples in the solid state at room temperature by serial electron diffraction using formvar encapsulation for sample preservation.

## 1. Introduction

The two main sources for producing diffraction patterns are X-rays and electrons. Depending on the experiment, one source may be preferable to the other. A key distinction between X-ray and electron crystallography lies in the sample requirements. Electron diffraction using transmission electron microscopy (TEM) offers a convenient tabletop method for crystal structure determination. However, challenges in sample preparation have historically limited its ability to achieve structural resolutions comparable to those obtained with X-ray techniques.

Structural analysis using TEM typically requires the specimen to be maintained under high-vacuum conditions, often in the range of 10^−5^ to 10^−6^ Pa, to minimize electron scattering by air molecules (Ayache *et al.*, 2009 ▸). This requirement restricts TEM analysis to organic or inorganic samples with very low vapor pressures. Samples such as anthracene and pyrene with high vapor pressures, when introduced into the microscope, tend to sublimate under high vacuum, resulting in detrimental effects on both the sample and the column vacuum of the electron microscope (Levin *et al.*, 2017 ▸). Cryo-methods involving rapid plunging of crystals into liquid ethane/nitro­gen solve this problem, but they require additional cryo-shielding to prevent ice build-up on the sample and greatly limit sample exchange, increasing costs and time for sample exchange; there is in addition the problem of not being able to sufficiently sample crystals of uniform thickness (Weis *et al.*, 2022 ▸; Gyobu *et al.*, 2004 ▸). This approach also limits the temperature range available for investigating thermally driven structural and phase changes.

Liquid-phase electron microscopy (LPEM), a complementary technique to cryo-electron microscopy, employs various sample sealing methods to enable in-liquid analysis of soft biological samples and their dynamics (Wu *et al.*, 2020 ▸; Kong *et al.*, 2023 ▸). Significant progress has been made in sealed liquid sample preparation for room-temperature (RT) LPEM using silicon nitride (SiN) windows, graphene-coated SiN windows and graphene liquid cells. However, sample sealing using SiN cells or graphene encapsulation typically requires a sophisticated process and custom fabrication with accompanying higher costs (Cho *et al.*, 2017 ▸; Yuk *et al.*, 2012 ▸; Williamson *et al.*, 2003 ▸; Plana-Ruiz *et al.*, 2023 ▸).

While significant advances have been made in sample protection methods for LPEM, a simple and widely applicable technique for preventing the sublimation of high-vapor-pressure organic crystalline samples under high vacuum at RT remains largely unexplored. Formvar (polyvinyl formal), a polymer widely used as a support film on TEM grids, is known for its electron transparency, radiation resistance, mechanical robustness and chemical inertness (Shukla *et al.*, 2000 ▸; Stadermann *et al.*, 2012 ▸; Auchter *et al.*, 2018 ▸; Callinan, 1951 ▸). Although typically used as a passive support, we demonstrate here that formvar can also act as an effective encapsulant, preserving ultrathin vacuum-sensitive organic crystals for RT electron diffraction (ED) studies.

In parallel, serial electron diffraction (SerialED) has emerged as a powerful method for structure determination of small crystals, particularly beam-sensitive samples. Unlike MicroED or 3D-ED approaches, where repeated exposure of a single crystal during a tilt series can lead to cumulative radiation damage (Jones *et al.*, 2018 ▸), SerialED collects diffraction patterns ideally from a large number of randomly oriented nanocrystals, each exposed to a single shot of the electron beam at low dose. The short exposure can be divided into multiple image fractions on the detector to monitor potential radiation damage. This strategy minimizes radiation damage by distributing the dose across many crystals and enabling dose fractionation, thereby allowing high-resolution structure determination. Furthermore, SerialED reduces the required sample quantity by several orders of magnitude (up to 10^4^ times less than for conventional single-crystal X-ray diffraction), making it ideal for precious or difficult-to-synthesize materials.

In this study, we report the successful structure determination of vacuum-sensitive organic compounds in their solid phase at room temperature using SerialED. By combining SerialED with a simple formvar encapsulation technique to protect samples from vacuum-induced sublimation, we demonstrate sub-Å resolution structure determination of anthracene and pyrene. This approach enables high-resolution structure determination of vacuum-sensitive, dry crystalline materials at RT and opens a new path for studying structural analysis and phase behavior without the constraints of cryogenic methods.

## 2. Materials and methods

### 2.1. Crystallization

The original compounds of anthracene, pyrene, formvar [poly(vinyl formal) ∼1% in 1,2-di­chloro­ethane] and organic solvents were purchased from Sigma–Aldrich. Anthracene was dissolved in cyclo­hexane by heating the solution to 60°C under constant stirring for 2 h. The solution was cooled to RT (25°C) and stirred for another 4 h. The resulting solution was filtered with a polyvinyl­idene fluoride filter of 0.2 µm pore size. Then, 5 µl of filtered saturated solution of anthracene in cyclo­hexane was drop-cast onto a 400-mesh copper grid with both continuous and lacey ultrathin carbon support film. Formvar film was wrapped, on the shiny side, over the sample to preserve it, effectively sandwiching the anthracene between the formvar and the carbon film.

Pyrene was dissolved in pentene, and the crystals formed at RT by slow solvent evaporation were sectioned to 100 nm sheets using a Leica Ultracut Ultramicrotome equipped with a PELCO diamond knife. The diamond knife trough was filled with a saturated aqueous solution, similarly to our previously published method (Daoud *et al.*, 2024 ▸). The sections were placed on the shiny side of a 400-mesh copper grid with continuous ultrathin carbon support film. Formvar film was cast over the shiny side to protect the sample.

### 2.2. TEM and ED measurements

ED measurements were performed using a Hitachi HT7800 transmission electron microscope operated at an accelerating voltage of 90 kV and recorded on an X-Spectrum Amber 750K hybrid pixel direct electron detector. TEM micrographs were acquired using a Hitachi HT7700 or HF3300 transmission electron microscope operated at 100 kV. The diffraction patterns were collected in scanning transmission electron microscopy (STEM) mode from various locations on the sample in a serial manner using Hitachi’s *Azorus* software package. First, an overview image of the sample was acquired in STEM mode under low-dose conditions. Second, a map of crystal positions was generated from the overview image. In this case, since the crystals were large thin sheets, the map took the form of a rectangular grid. Finally, *Azorus* used the map coordinates to automatically move the electron probe to each position and capture the diffraction patterns. The approximate electron beam sizes for diffraction acquisition were 250 nm for anthracene and 170 nm for pyrene. The electron probe used for both samples was nearly parallel, with a convergence angle of ∼0.2 mrad, although slight variations may occur between anthracene and pyrene due to differences in beam size. The overview STEM image can be obtained using a smaller probe (nanometre-sized) for image clarity, and then the probe can be expanded to the desired size for diffraction acquisition. However, sharp STEM images are not usually required, and as long as the crystals are distinguishable, the overview STEM image can be collected with the same probe as used for diffraction acquisition for simplicity.

Annular dark field (ADF)-STEM images were collected using a ∼20 nm probe with a resolution of 512 × 512 pixels for both samples and a dwell time of 7.2 µs. This exposure corresponds to an electron fluence of ∼0.01 e Å^−2^, which is only ∼1% of the fluence used for diffraction acquisition (∼1 e Å^−2^), indicating that beam damage during overview imaging is negligible. For anthracene, different fields of view (FOVs) were selected to match the crystal size, ranging from 2.9 × 2.9 µm to 6.4 × 6.4 µm, with data collected from approximately 150 sample regions. For pyrene, a consistent FOV of 3.3 × 3.3 µm was used, with data collected from approximately 130 sample regions. The sample was moved manually between regions, while crystal selection was carried out automatically using a thresholding algorithm. An example of ADF-STEM overview images for anthracene and pyrene, along with the selected diffraction points, is shown in Fig. S1 in the supporting information. To monitor the beam damage in the samples, multiple frames were recorded at each crystal position, and the frames were cut during the data analysis step when the high-resolution diffraction spots started to fade. For anthracene, six diffraction frames were aggregated for each crystal position, with an exposure time of 15 ms per frame, resulting in a total exposure time of 90 ms per crystal. The electron fluence was calculated by measuring the beam current using a Faraday cup. The total fluence per crystal position was 1.0 e Å^−2^, which is well below the damage threshold for most beam-sensitive materials (Egerton *et al.*, 2004 ▸; Egerton, 2019 ▸). The diffraction patterns collected from different positions of a crystal were uniform, and to capture patterns from different orientations of the crystalline thin sheets, the sample was tilted slightly (up to ±45°) when moving from one crystal region to another. For pyrene, four diffraction frames of 15 ms each were aggregated, corresponding to a total electron fluence of 1.3 e Å^−2^ per crystal.

The diffraction data were processed using the *diffractem* package (Bücker *et al.*, 2021 ▸) and the *CrystFEL* software (White *et al.*, 2012 ▸). The diffraction peaks were located by *CrystFEL* using the *Peakfinder9* algorithm. The *diffractem* package was then used to find the center of each pattern and correct the elliptical distortion in the diffraction images. The anthracene data exhibited an elliptical distortion with an eccentricity of 1.023, whereas this distortion was negligible for the pyrene data after careful alignment of the microscope in the diffraction setting. The data were then indexed by *CrystFEL* using the *Pinkindexer* algorithm (Gevorkov *et al.*, 2020 ▸).

The structure solution was performed by *SHELXT* (Sheldrick, 2015 ▸) and subsequent structure refinement and visualization with *Olex2* (Dolomanov *et al.*, 2009 ▸). The outlier reflections were identified from the observed versus calculated reflections plot (*F*_o_ versus *F*_c_) during the structure refinement using *Olex2* and were removed to enhance structure quality (Cichocka *et al.*, 2018 ▸).

## 3. Results and discussion

### 3.1. Sample sealing using formvar

**Figure 1:**
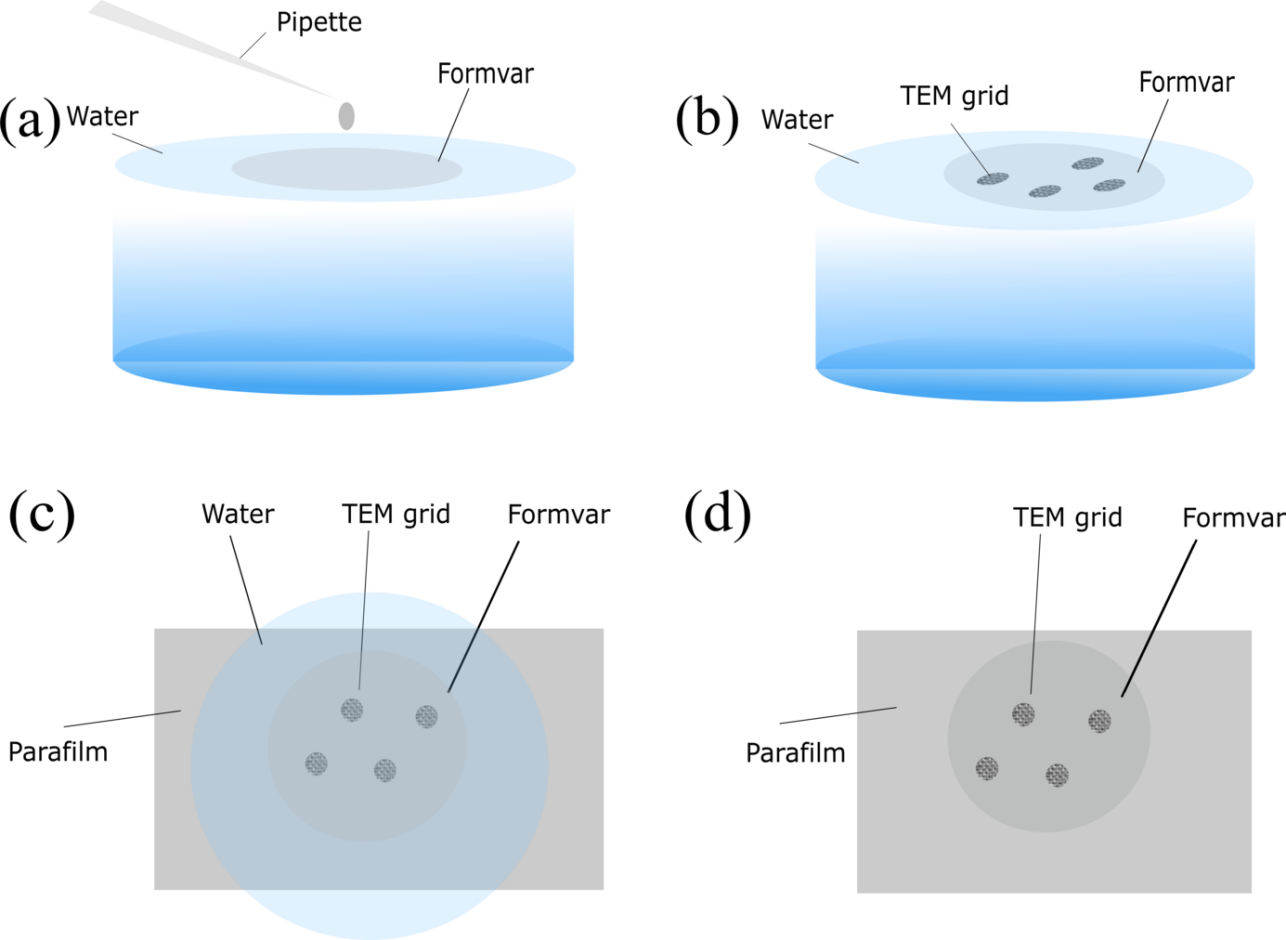
Step-by-step schematic of the formvar covering process. (*a*) Circular formvar film formed by the drop-cast method. (*b*) Multiple sample holding grids placed over the formvar film with the sample side facing the formvar film. (*c*) Top view after lowering parafilm to lift the formvar along with the grids. (*d*) Formvar-adhered parafilm kept for drying with parafilm at the bottom and formvar at the top.

Formvar films were prepared by drop-casting a formvar suspension on an air–water surface as described by Davison & Colquhoun (1985 ▸). The schematic of the formvar covering process is given in Fig. 1 ▸, and photographs of the step-by-step experimental procedure are given in Fig. S2. Nearly 3 µl of formvar suspension was taken in a micropipette and dispensed onto the surface of water from a height of less than 1 mm above the water surface to form circular formvar films, as represented by the schematic Fig. 1 ▸(*a*). The sample holding grids were placed well separated on the circular formvar film with the shiny sample side down, facing the formvar film as in Fig. 1 ▸(*b*). A fresh parafilm was lowered slowly to pick up this circular film enclosing grids from the top [Fig. 1 ▸(*c*)]. The formvar films cast by this method adhered to the parafilm very well, and multiple grids were covered in a single batch [Fig. 1 ▸(*d*)], considerably reducing sample preparation time and effort. The parafilm was left to dry, and the sides of the grids were scratched with sharp tweezers normal to the outer surface of the grid. As the formvar covering is performed directly over the sample, encapsulation is achieved using a single grid. A cross-sectional view of the grid and the sample encapsulation is shown in Fig. S3.

The samples successfully wrapped with formvar film survived under the high-vacuum TEM conditions for several hours without sublimation artifacts. A complete ED dataset was acquired for anthracene and pyrene at RT. The structures were successfully solved, as detailed in the following section.

### 3.2. Structure determination

Previous ED studies on anthracene were performed at low temperatures (Skrebowski, 1948 ▸). A control sample of anthracene was prepared by the drop-cast method, and these samples were sublimated within 100 s after loading the grid into the vacuum column at RT. TEM images showing the sublimation effects are given in Fig. S4. A video capture of the sublimation process at RT was recorded and is included in the supporting information. As the pyrene samples used in this study were 100 nm sections, the control sample readily sublimated, and the sample sheets no longer existed upon alignment. In this work, we performed SerialED at RT on formvar-covered anthracene crystals formed by drop-casting and obtained a high-quality dataset that enabled high-resolution structure determination. The sample remained stable during the data collection, which took a few hours to complete. Over 9400 diffraction patterns were indexed to determine the Miller indices (*h*, *k*, *l*) and the intensities of the diffraction spots. The quality of diffraction changes dramatically for anthracene at RT without the formvar coating, where no discernible diffraction was observed.

**Figure 2:**
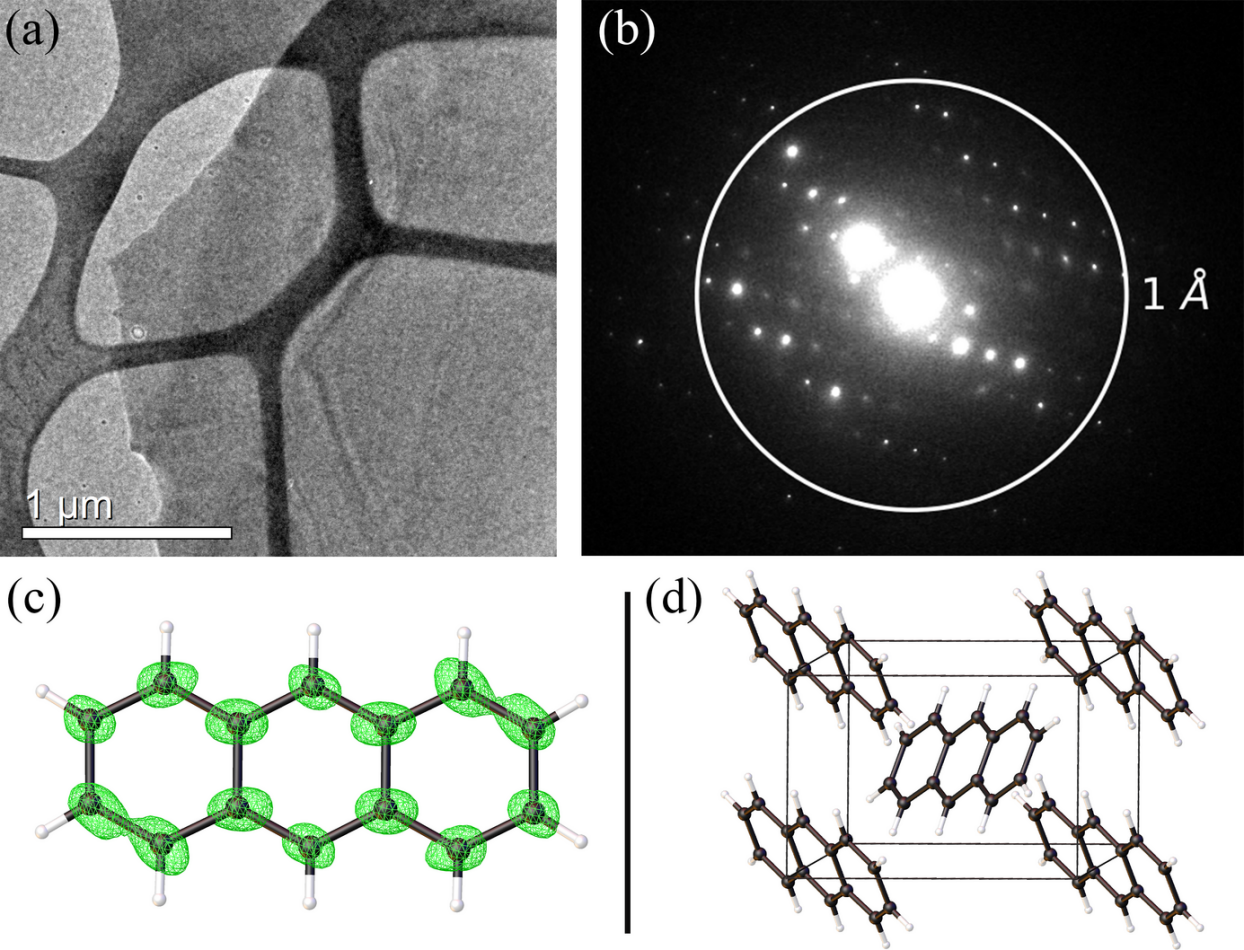
(*a*) TEM image of a thin anthracene crystal encapsulated with formvar. (*b*) Electron diffraction pattern showing well defined diffraction spots extending beyond the 1.0 Å resolution ring. (*c*) Solved structure of anthracene obtained from SerialED data, overlaid with the electron-density map (2*F*_o_ − *F*_c_) around carbon and hydrogen atoms. (*d*) Molecular packing of anthracene in the crystal structure.

Fig. 2 ▸(*a*) shows a TEM image of a thin anthracene crystal sheet covered with formvar. A representative ED pattern is shown in Fig. 2 ▸(*b*), with diffraction spots extending well beyond the 1.0 Å resolution ring. The structure of anthracene was solved using SerialED data collected from this sample. The final model is presented in Fig. 2 ▸(*c*), along with the electron-density map (2*F*_o_ − *F*_c_) around the atoms. As expected, anthracene consists of three linearly fused benzene rings.

We achieved a resolution of 0.75 Å for this structure. The resolution was determined from the cross-correlation coefficient CC* or CC_1/2_, and the data were truncated when CC* dropped below 0.5 (or CC_1/2_ below 0.143) (Karplus & Diederichs, 2012 ▸). The molecular packing of anthracene in the crystal is depicted in Fig. 2 ▸(*d*), and it agrees well with previously reported X-ray crystallography structures (Lusi *et al.*, 2015 ▸).

**Figure 3:**
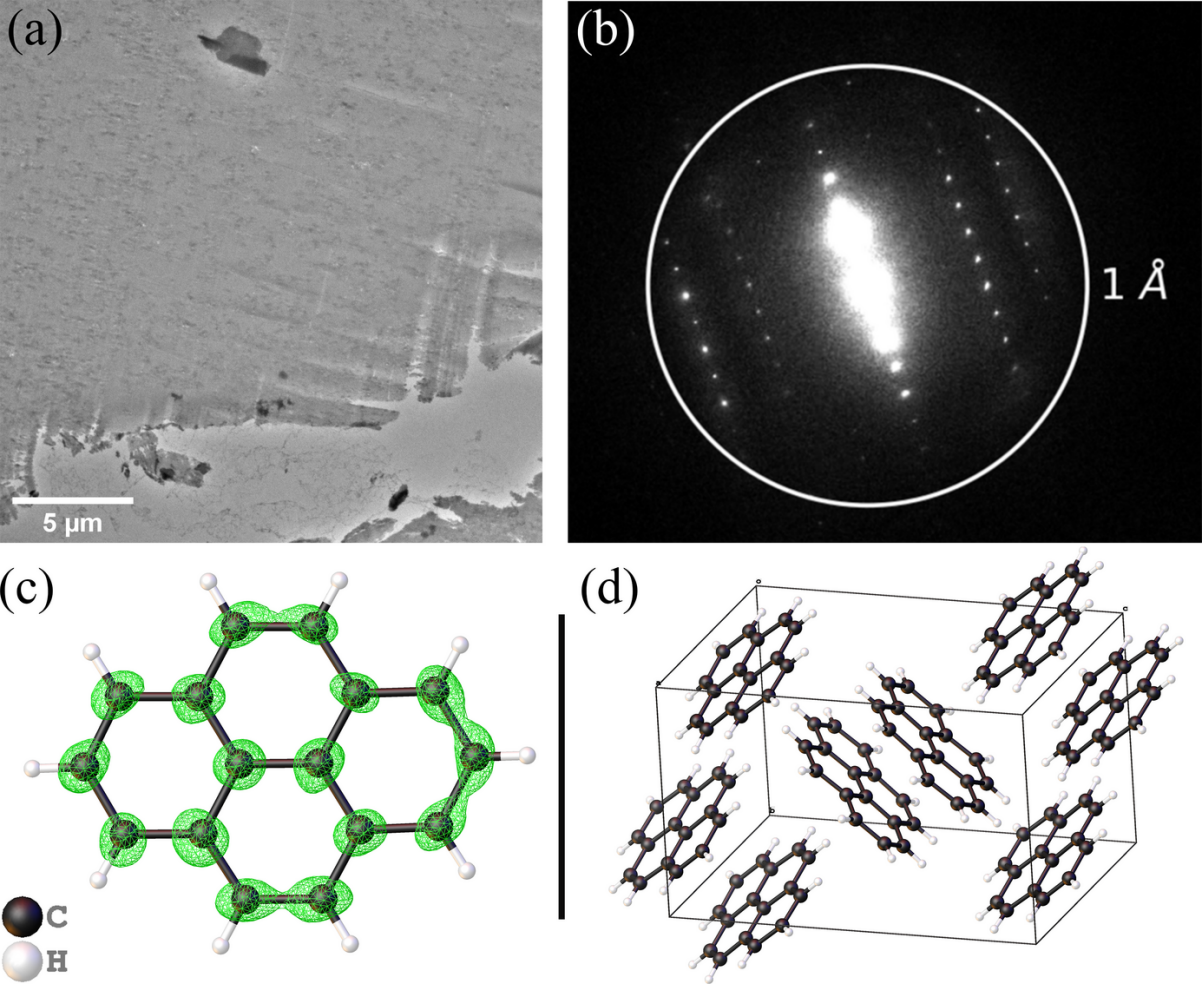
(*a*) TEM image of thin pyrene crystals encapsulated with formvar. (*b*) Representative electron diffraction pattern of pyrene, showing strong high-resolution peaks. (*c*) Solved structure of pyrene from SerialED data, overlaid with the electron-density map (2*F*_o_ − *F*_c_). (*d*) Molecular arrangement of pyrene within the unit cell.

The structure of pyrene was similarly determined using SerialED. Approximately 3100 indexed crystals contributed to a final resolution of 0.80 Å. A TEM image of the pyrene crystal section covered with formvar is shown in Fig. 3 ▸(*a*). A representative diffraction pattern is shown in Fig. 3 ▸(*b*), highlighting strong peaks at high resolution. The solved structure, overlaid with the electron-density map, is presented in Fig. 3 ▸(*c*), and the molecular arrangement within the unit cell is shown in Fig. 3 ▸(*d*).

Crystallographic parameters and structure refinement details for anthracene and pyrene are summarized in Table 1 ▸. The structure solutions for both samples were performed at 90 kV, which results in significant dynamical scattering compared with the standard 200 kV typically used in electron microscopy and diffraction measurements. The lower electron energy reduces knock-on beam damage to the sample and can potentially enable higher-resolution structures compared with higher beam energies (Egerton, 2019 ▸). However, dynamical scattering is increased at these lower energies (Carter & Williams, 2016 ▸), which can lead to higher *R* factors and less favorable refinement statistics. Precession ED can be employed to mitigate dynamical scattering (Plana-Ruiz *et al.*, 2025 ▸), and dynamical refinement can further reduce its effects during data analysis, ultimately improving structure solution and refinement quality (Clabbers *et al.*, 2019 ▸).

## 4. Conclusion

We have demonstrated that SerialED enables high-resolution structure determination of vacuum-sensitive organic compounds in their solid phase at room temperature, without the need for cryogenic protection. By distributing the electron dose across the sample, SerialED overcomes the limitations of beam-induced damage that typically restrict conventional ED studies, and it achieves sub-Å resolution even for beam-sensitive and vacuum-sensitive materials.

As a critical enabling step, we employed a simple formvar encapsulation technique to prevent sample sublimation under high vacuum, allowing SerialED to be applied to vacuum-sensitive organic crystals. Using this approach, we successfully solved the structure of anthracene at 0.75 Å resolution and pyrene at 0.80 Å. Importantly, the sample preparation method relies on readily available materials and does not require specialized or expensive instrumentation, making it widely accessible. This sample encapsulation method is suitable not only for SerialED but also for rotation-based ED techniques such as MicroED. In the future, this encapsulation approach may enable room-temperature MicroED measurements of similar vacuum-sensitive organic crystals as well.

This combined method – SerialED paired with formvar encapsulation – offers the potential of a rapid and accessible workflow for high-resolution structure determination. Sample preparation can be completed in minutes, data acquisition in a few hours, and data analysis can be performed in parallel, significantly accelerating the overall process. This strategy enables room-temperature structure determination with minimal compromise in resolution and offers potential for uncovering structure–function relationships that require room temperature or temperatures above cryogenic conditions to faithfully represent the process of interest.

Furthermore, SerialED significantly reduces the amount of material required for structure analysis, making it particularly well suited for precious or hard-to-synthesize compounds. Because the method relies on standard TEM hardware, it is broadly accessible and can be implemented routinely in structural characterization laboratories.

In summary, this work establishes SerialED combined with formvar encapsulation as a powerful and practical method for high-resolution structure determination of vacuum- and beam-sensitive materials at room temperature, with the potential to transform the way structural studies are performed across materials science and chemistry.

## Supplementary Material

Crystal structure: contains datablock(s) anthracene_a2, pyrene_a2. DOI: 10.1107/S1600576725009823/hat5012sup1.cif

ADF-STEM images with selected points for diffraction acquisition, photographs of the step-by-step experimental procedure of formvar sealing, cross-sectional view of the sample encapsulation and images showing the sublimation of anthracene without formvar encapsulation under TEM vacuum conditions at RT. DOI: 10.1107/S1600576725009823/hat5012sup2.pdf

A video showing the sublimation of anthracene samples (without formvar encapsulation) under the vacuum conditions of TEM at RT. DOI: 10.1107/S1600576725009823/hat5012sup3.mp4

CCDC references: 2464360, 2479753

## Figures and Tables

**Table 1:** Crystallographic details of the structure solution and refinements of anthracene The values in parentheses represent the statistics at the highest-resolution shell.

	Anthracene	Pyrene
Formula	C_14_H_10_	C_16_H_10_
Space group	*P*2_1_/c	*P*2_1_/c
Unit-cell lengths *a*, *b*, *c* (Å)	9.50, 6.00, 8.55	8.47, 9.26, 13.65
Unit-cell angles α, β, γ (°)	90.0, 103.74, 90.0	90.0, 100.28, 90.0
Temperature (K)	298	298
Electron wavelength (Å)	0.03919	0.03919
Resolution (Å)	9.23–0.75 (0.78–0.75)	13.4–0.80 (0.83–0.80)
Completeness (%)	82.3 (78.1)	93.2 (85.7)
CC_1/2_	98.6 (66.1)	93.1 (33.9)
CC*	99.6 (89.2)	98.2 (71.2)
Unique reflections	950	2015
*I*/σ(*I*)	5.67 (2.57)	2.98 (1.12)
Redundancy	96.4 (43.2)	30.3 (12.1)
*R*_split_	0.13 (0.46)	0.25 (0.91)
Goodness of fit	2.324	1.65
*R*1, *wR*2 (all data)	0.286, 0.601	0.290, 0.575
*R*1 [*F* > 4σ(*F*)]	0.264	0.243
