# Supplementary material for: Room-temperature structure determination of vacuum-sensitive organic compounds by formvar encapsulation and serial electron diffraction
Source: J Appl Crystallogr. 2025 Nov 26;58(Pt 6):2119–24. doi: 10.1107/S1600576725009823 (PMC12810524; doi:10.1107/S1600576725009823)
Supplement: Supplementary file 2 [file j-58-02119-sup2.pdf]

JOURNAL OF
APPLIED
CRYSTALLOGRAPHY

Volume 58 (2025)

Supporting information for article:

Room-Temperature Structure Determination of Vacuum-Sensitive Organic Compounds by Formvar Encapsulation and Serial Electron Diffraction

Sreelaja Pulleri Vadhyar, Ehsan Nikbin, Hazem Daoud, Jane Y. Howe and R. J. Dwayne Miller

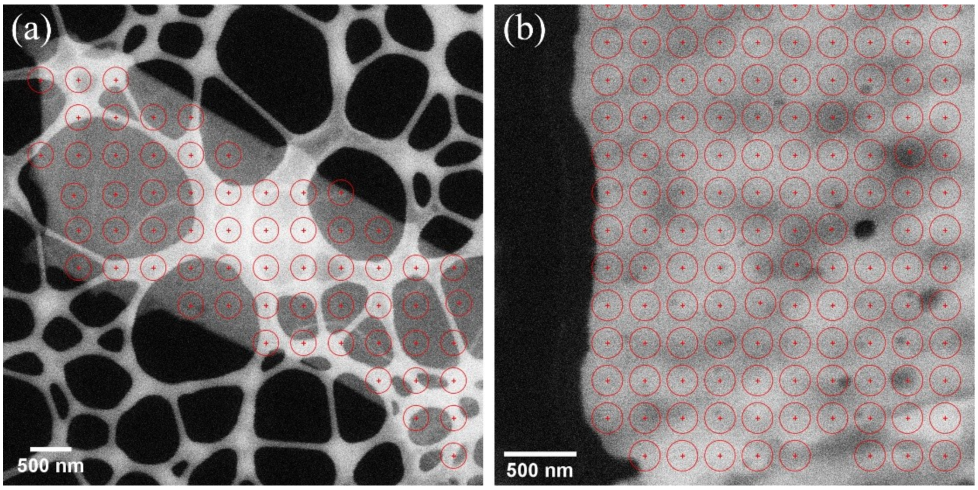

Figure S1 Examples of ADF-STEM images with selected points for diffraction acquisition. Diffraction points were identified using an automatic thresholding algorithm and are marked by red circles; the circle diameter corresponds to the probe size. (a) Image of an anthracene crystal with a field of view of 5.75 × 5.75 µm². The probe size was ~250 nm with a spacing of ~450 nm. (b) Image of a pyrene crystal with a field of view of 3.30 × 3.30 µm². The diffraction probe size was ~170 nm with a spacing of ~260 nm.

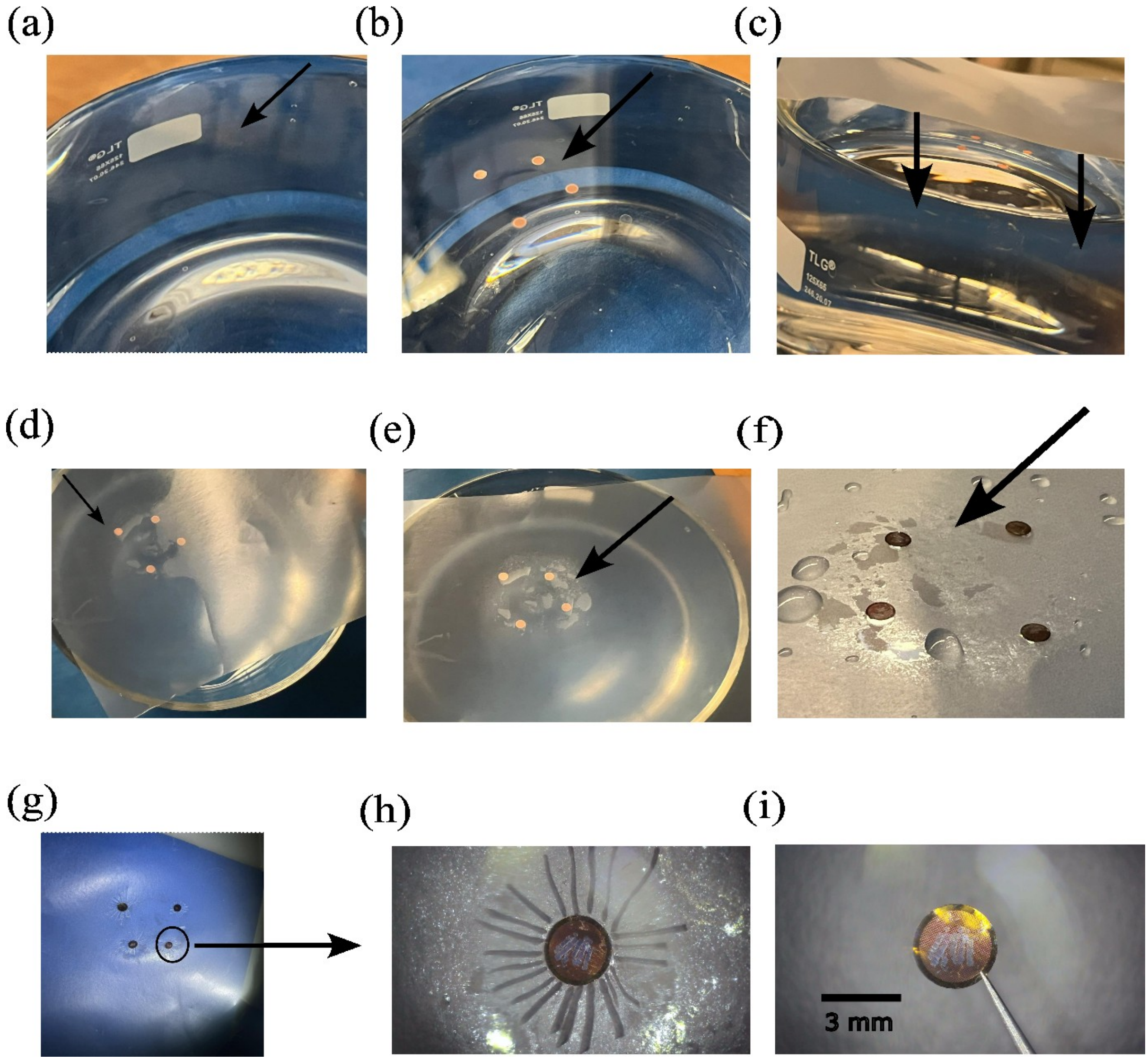

Figure S2 Photographs of the formvar covering process. (a) Circular formvar film (indicated by black arrow) formed by drop cast method (b) Four grids with 100 nm thin sample sections, placed over formvar film (indicated by black arrow) with the sample side facing the formvar film (c) Lateral view of parafilm approaching the grids with the black arrows indicating the direction of motion of parafilm towards formvar film (d) Photograph during the process of adhesion of Formvar to parafilm. Arrow indicates the grid securely encapsulated between the parafilm and formvar (e) A circular formvar film completely adhered to parafilm, ready for lifting. (f) The arrow indicates the Formvar film successfully lifted from the water and kept for drying. (g) The periphery of each grid is scratched to separate the formvar covered sample grid from parafilm. (h) A clear image of scratch lines around the TEM grid before separating the grid from parafilm. (i) Magnified image of a separated TEM grid with an intact sample sheet, covered with formvar film.

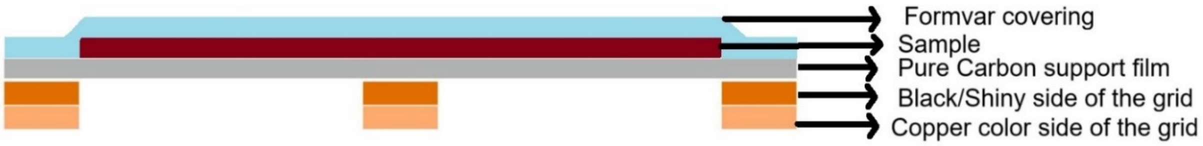

Figure S3 Cross-sectional view of the sample encapsulation. A TEM grid with a pure carbon layer (or pure carbon with lacey underneath) was used to encapsulate the sample between the carbon layer and the Formvar covering layer to avoid sample sublimation in high-vacuum.

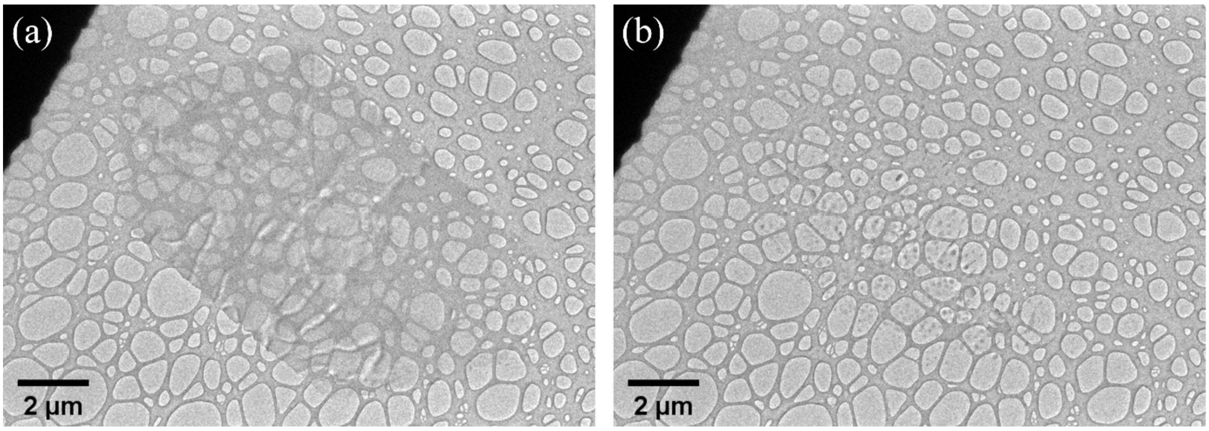

Figure S4 Sublimation of anthracene without formvar encapsulation under TEM vacuum conditions. (a) TEM image of an anthracene crystal (without formvar encapsulation) acquired shortly after sample insertion. (b) TEM image acquired after 100 s, showing that the crystal has sublimated. The electron beam was blanked between the two acquisitions.
